# Cardiac α_V_β_3_ integrin expression following acute myocardial infarction in humans

**DOI:** 10.1136/heartjnl-2016-310115

**Published:** 2016-12-07

**Authors:** William S A Jenkins, Alex T Vesey, Colin Stirrat, Martin Connell, Christophe Lucatelli, Anoushka Neale, Catriona Moles, Anna Vickers, Alison Fletcher, Tania Pawade, Ian Wilson, James H F Rudd, Edwin J R van Beek, Saeed Mirsadraee, Marc R Dweck, David E Newby

**Affiliations:** 1 British Heart Foundation Centre for Cardiovascular Science, University of Edinburgh, Edinburgh, UK; 2 Clinical Research Imaging Center, University of Edinburgh, Edinburgh, UK; 3 Edinburgh Molecular Imaging Ltd, Edinburgh, UK; 4 Division of Cardiovascular Medicine, University of Cambridge, Cambridge, Cambridgeshire, UK

## Abstract

**Objective:**

Maladaptive repair contributes towards the development of heart failure following myocardial infarction (MI). The α_v_β_3_ integrin receptor is a key mediator and determinant of cardiac repair. We aimed to establish whether α_v_β_3_ integrin expression determines myocardial recovery following MI.

**Methods:**

^18^F-Fluciclatide (a novel α_v_β_3_-selective radiotracer) positron emission tomography (PET) and CT imaging and gadolinium-enhanced MRI (CMR) were performed in 21 patients 2 weeks after ST-segment elevation MI (anterior, n=16; lateral, n=4; inferior, n=1). CMR was repeated 9 months after MI. 7 stable patients with chronic total occlusion (CTO) of a major coronary vessel and nine healthy volunteers underwent a single PET/CT and CMR.

**Results:**

^18^F-Fluciclatide uptake was increased at sites of acute infarction compared with remote myocardium (tissue-to-background ratio (TBR_mean_) 1.34±0.22 vs 0.85±0.17; p<0.001) and myocardium of healthy volunteers (TBR_mean_ 1.34±0.22 vs 0.70±0.03; p<0.001). There was no ^18^F-fluciclatide uptake at sites of established prior infarction in patients with CTO, with activity similar to the myocardium of healthy volunteers (TBR_mean_ 0.71±0.06 vs 0.70±0.03, p=0.83). ^18^F-Fluciclatide uptake occurred at sites of regional wall hypokinesia (wall motion index≥1 vs 0; TBR_mean_ 0.93±0.31 vs 0.80±0.26 respectively, p<0.001) and subendocardial infarction. Importantly, although there was no correlation with infarct size (r=0.03, p=0.90) or inflammation (C reactive protein, r=−0.20, p=0.38), ^18^F-fluciclatide uptake was increased in segments displaying functional recovery (TBR_mean_ 0.95±0.33 vs 0.81±0.27, p=0.002) and associated with increase in probability of regional recovery.

**Conclusion:**

^18^F-Fluciclatide uptake is increased at sites of recent MI acting as a biomarker of cardiac repair and predicting regions of recovery.

**Trial registration number:**

NCT01813045; Post-results.

## Introduction

Ischaemic heart disease remains the leading cause of death globally, with over 1 million people suffering acute myocardial infarction (MI) per year in the USA alone. As the acute management of MI improves, the number of patients surviving acute myocardial injury is higher than ever before. In this population, adverse cardiac remodelling and the syndrome of delayed heart failure represent the major cause of morbidity.^1^ Understanding reparative mechanisms following infarction is becoming increasingly important.

Repair following MI is triggered by a complex interaction of neurohormonal activation and upregulation of angiogenic and pro-fibrotic transcription factors that initiate the restoration of a capillary network through angiogenesis and re-endothelialisation, as well as extracellular matrix (ECM) remodelling through macrophage accumulation and fibroblast activation. This interplay of angiogenesis, inflammation and fibrosis determines the extent of preservation and restoration of myocardial integrity.^2^ In some circumstances, maladaptive persistent processes may encourage remodelling and scarring to extend into the myocardium long after the initial causative injury. This may lead to progressive ventricular dilatation, ventricular dysfunction and heart failure.

The α_v_β_3_ integrin is a transmembrane cell surface receptor that facilitates migration, proliferation and interaction with the ECM, thereby allowing cells to respond to, and in turn modify, their extracellular environment. Expressed at low levels by quiescent endothelial cells, α_v_β_3_ integrin is markedly upregulated in states of angiogenesis within the myocardium after infarction.^3^ ^4^ In addition, preclinical and clinical studies document α_v_β_3_ integrin expression by both activated cardiac myofibroblasts and macrophages during margination and chemotaxis. Thus, α_v_β_3_ integrin expression appears central to the coordination of repair following MI.

In this study, we investigated the expression of α_v_β_3_ integrin following MI using the novel α_v_β_3_ integrin-selective radiotracer, ^18^F-fluciclatide, combined with cardiac positron emission tomography (PET), CT and cardiovascular MRI (CMR). The study aims to describe and characterise the uptake of this radiotracer and to correlate it with clinical markers of disease severity and functional recovery in patients with recent MI.

## Methods

PET/CT scanning with ^18^F-fluciclatide and CMR were performed in three groups of participants recruited from Royal Infirmary of Edinburgh between July 2013 and February 2015. Exclusion criteria were age <40 years, women of childbearing potential not taking contraception, severe renal failure (serum creatinine >2.8 mg/dL) or hepatic failure (Child-Pugh grade B or grade C), atrial fibrillation, contrast allergy, inability to undergo scanning and inability to provide informed consent. Studies were performed with approval of the local research ethics committee, in accordance with the Declaration of Helsinki, and with the written informed consent of each participant.

### Study participants

#### Acute MI group

Patients with recent acute ST-segment elevation MI and peak high-sensitivity cardiac troponin I (hs-cTnI) >10 000 ng/L were invited to attend PET/CT scanning with ^18^F-fluciclatide 14±7 days after their initial presentation. CMR was performed within 7 days of PET/CT scanning. Patients were then invited to return for a second PET/CT scan with ^18^F-fluciclatide 10 weeks after MI and for a follow-up CMR at 9 months.

#### Chronic total occlusion group

Patients with an angiographically documented complete occlusion of a major epicardial artery and stable cardiac symptoms for >6 months were invited to attend for a single PET/CT scan with ^18^F-fluciclatide and CMR.

#### Control group

Volunteers with normal left ventricular (LV) systolic function, no structural heart disease and no symptoms of heart failure or MI underwent a single PET/CT scan with ^18^F-fluciclatide and CMR.

#### Histology cohort

For histological analysis, myocardial biopsy samples were obtained from patients undergoing coronary artery bypass grafting following recent MI. Patients with large ST-elevation MI (<14 days, hs-cTnI >10 000 ng/L) were considered for inclusion, forming a separate cohort from the acute MI group.

### Radiosynthesis of ^18^F-fluciclatide

The radiotracer was manufactured at the Clinical Research Imaging Centre, University of Edinburgh on an automated module (FASTlab synthesizer; GE Healthcare) by coupling an amino-oxy-functionalised peptide precursor (AH111695) with 4-^18^F-fluorobenzaldehyde at pH 3.5 to form ^18^F-fluciclatide. Full description of this synthesis has been published.^5–7^

### Imaging assessments

All patients underwent PET-CT imaging of the thorax with a hybrid scanner (Biograph mCT, Siemens Medical Systems, Germany) after administration of a target dose of 230 MBq ^18^F-fluciclatide (see online supplementary material). No dietary restrictions were required prior to radiotracer administration. Attenuation-correction CT scanning (non-enhanced 120 kV and 50 mA, 3 mm slices) was performed, followed by PET acquisition with ECG gating. To assess tracer pharmacodynamics and the optimum timing of scanning, dynamic thoracic PET imaging was initially performed in 10 subjects in three-dimensional mode with a single-bed position for 70 min. The remainder of study subjects underwent static imaging performed at the optimal time point as determined from the dynamic studies (40 min post-injection) using a single 30 min bed position in list mode. Immediately after PET acquisition, cardiac CT angiography was performed on the hybrid scanner (see online supplementary material).

10.1136/heartjnl-2016-310115.supp1 supplementary data

### PET reconstruction and analysis

Kinetic analysis of the dynamic scans was undertaken to investigate ^18^F-fluciclatide uptake within the myocardium (see online supplementary material). For all patients, static ECG-gated PET images were reconstructed in diastole, 40–70 min post-injection. Myocardial ^18^F-fluciclatide uptake was assessed by an experienced observer (WSAJ) by drawing regions of interest (ROIs) in each myocardial segment using the standardised 16-segment approach.^8^ Additionally, ROIs were drawn in focal regions affected by infarction and in regions of remote myocardium. This was achieved using the fused CT angiography and CMR images as a reference and with care taken to avoid contamination from the blood pool signal. PET data were corrected for residual blood pool activity (standard uptake value, SUV) in the superior vena cava and expressed as a mean tissue-to-background ratio (TBR_mean_). SUV_max_ and TBR_max_ values were also calculated alongside the corrected SUV values, where blood pool activity was subtracted from myocardial uptake (see online supplementary material).

### CMR imaging

CMR with assessment of late gadolinium enhancement (LGE) and T1 mapping was performed at 3 T (MAGNETOM Verio, Siemens AG, Healthcare Sector, Germany) with calculation of LV function, wall motion index (WMI), transmurality of LGE and extracellular volume (ECV) fraction (see online supplementary material).

### Histology

Briefly, a core cardiac biopsy was taken from the peri-infarct zone, fresh-frozen and sectioned in cryosection medium (see online supplementary material). Adjacent tissue sections were stained with HE for conventional, smooth muscle actin, CD31, CD68 (clone PG-M1) and integrin α_v_β_3_ antibody clone LM609 (Millipore) before digital imaging (Axioscan.Z1, Zeiss, UK) and histopathological examination.

### Statistical analysis

We explored myocardial radiotracer uptake in three groups of patients, comparing them with CMR and clinical indices of cardiac function. Continuous data were tested for normality with the D'Agostino and Pearson Omnibus test. Continuous normal variables were expressed as mean±SD and compared using Student’s t-test or analysis of variance test when comparing more than two groups. Continuous non-normal variables were presented as median (IQR) and compared using the Mann-Whitney or Kruskal-Wallis test when evaluating two or more than two groups. Interobserver reproducibility was calculated by Bland-Altman method and presented as mean bias ±2 SDs and intraclass correlation coefficients (ICC) with 95% CI. The χ^2^ test was used for analysis of categorical variables. Univariable and multivariable logistic regression models were used to determine factors associated with an improvement in segmental myocardial function. Statistical analysis was performed with GraphPad Prism (V.6; GraphPad Software, USA) and JMP (V.10.0; SAS Software, North Carolina, USA), where appropriate. A two-sided p value<0.05 was considered statistically significant.

## Results

A total of 37 subjects underwent PET/CT after injection of 229±12 MBq ^18^F-fluciclatide: 21 patients with acute MI, 7 patients in the chronic total occlusion (CTO) group and nine healthy volunteers (tables 1 and 2). The groups were generally well balanced for age, sex and body mass index. Healthy subjects had a lower prevalence of cardiovascular risk factors (table 1). The mean radiation dose per participant for those who received a single PET/CT imaging assessment was 13.6 mSv (range 7.8–18.9 mSv) and 21.9 mSv (range 16.5–28.7 mSv) in those who underwent repeat PET scanning.

**Table 1:** Baseline participant characteristics

	All (n=37)	Acute myocardial infarction group (n=21)	Chronic total occlusion group (n=7)	Control group (n=9)	p Value*
Patient characteristics					
Age (years)	64±10	62±12	69±7	66±7	0.06
Male sex	27 (73)	16 (76)	5 (71)	6 (67)	0.46
BMI (kg/m^2^)	28±4	28±5	31±3	27±4	0.74
^18^F-Fluciclatide dose (mBq)	229±12	227±13	227±14	232±9	0.86
Current smoker	9 (24)	8 (38)	1 (14)	0 (0)	0.01
Diabetes mellitus	4 (11)	3 (14)	1 (14)	0 (0)	0.06
Hypertension	16 (43)	7 (33)	5 (71)	4(44)	0.04
Hypercholesterolaemia	22 (60)	14 (66)	6 (86)	2 (22)	<0.001
Cardiovascular history					
Prior myocardial infarction	7 (19)	1 (5)	6 (86)	0 (0)	<0.001
Angiographically documented CAD	28 (76)	21 (100)	7 (100)	0 (0)	<0.001
Previous PCI	4 (11)	1 (5)	3 (42)	0 (0)	<0.001
CCS class					
0	28 (76)	17 (81)	2 (29)	9 (100)	0.02
I or II	7 (19)	4 (19)	3 (42)	0 (0)	0.03
III or IV	2 (6)	0 (0)	2 (29)	0 (0)	0.51
NYHA class					
I	29 (78)	15 (71)	3 (42)	9 (100)	0.32
II	7 (19)	5 (24)	2 (29)	0 (0)	0.39
III or IV	2 (6)	1 (5)	1 (14)	0 (0)	0.25
Medications					
Aspirin	27 (73)	21 (100)	6 (86)	0 (0)	<0.001
Clopidogrel	22 (59)	19 (90)	3 (42)	0 (0)	<0.001
Statin	29 (78)	21 (100)	7 (100)	1 (11)	0.004
β-blocker	26 (70)	20 (95)	6 (86)	0 (0)	<0.001
ACEi/ARB	27 (73)	20 (95)	5 (71)	2 (22)	0.02
Clinical features					
Systolic BP (mm Hg)	137±22	128±18	140±24	155±21	0.06
Heart rate (bpm)	64±12	62±13	61±11	68±10	0.42
Creatinine (µmol/L)	79±15	82±19	77±14	73±12	0.54
hs-CRP (mg/L)	3.5 (1.3–9.8)	5.6 (2.0–11.7)	2.2 (1.0–9.1)	1.5 (1.2–3.3)	0.008

*Analysis of variance, Student’s t-test (continuous data) or χ^2^ test (categorical data).

Mean±SD, median (IQR) or number (percentage).

ACEi, ACE inhibitor; ARB, angiotensin receptor blocker; BMI, body mass index; BP, blood pressure; CAD, coronary artery disease; CCS, Canadian Cardiovascular Society; hs-CRP, high-sensitivity C reactive protein; NYHA, New York Heart Association; PCI, percutaneous coronary intervention.

### CMR characterisation of MI and remodelling

There were no areas of infarction in control subjects. All patients within the acute MI cohort (n=21) had visible infarction on CMR 13±5 days after MI. These infarcts were large (infarct size 12±7 g/m^2^, peak cTnI 50 000 ng/L (26 753–50 000 ng/L)) and featured the anterior (n=16) and lateral (n=4) territories predominantly, while one patient had an inferior MI. All subjects received emergency coronary angiography with successful revascularisation 197 min (148–342 min) from the onset of symptoms (see online supplementary table S1).

Old infarcts were present in six of seven patients in the CTO group. Although there was no difference in infarct size (g/m^2^) between MI and CTO groups (p=0.34), the LV ejection fraction (LVEF) was reduced with a larger WMI score in those with recent MI (p<0.01 compared with either the CTO or control group, table 2).

**Table 2:** Baseline imaging assessment

	All (n=37)	Acute myocardial infarction group (n=21)	Chronic total occlusion group (n=7)	Control group (n=9)	p Value*
CMR imaging					
LVEF (%)	58±10	52±9	62±8	65±5	<0.001
LV mass (indexed, g/m^2^)	79±20	85±20	66±17	75±16	0.07
LVEDV (mL/m^2^)	77±18	80±18	69±13	76±17	0.35
LVESV (mL/m^2^)	34±13	39±14	30±10	27±9	0.046
WMI	0.25±0.13	0.40±0.20	0.09±0.05	0.0±0.0	<0.001
ECV (%)	31±5	34±4	28±3	28±2	<0.001
Presence of LGE	27 (73)	21 (100)	6 (86)	0 (0)	<0.001
Infarct size (g/m^2^)	8±8	12±7	8±8	0±0	<0.001
PET imaging					
SUV_mean_ (kBq/mL)					
SVC	2.73±0.51	2.85±0.51	2.57±0.39	2.58±0.57	0.27
Total LV uptake	2.24±0.51	2.33±0.48	1.96±0.53	1.77±0.27	<0.001
Myocardial infarct uptake	3.23±1.03	3.72±0.63	1.76±0.26	–	<0.001
Remote myocardial uptake	2.21±0.60	2.41±0.57	1.62±0.14	–	0.001
TBR_mean_					
Total LV uptake	0.77±0.16	0.82±0.18	0.71±0.06	0.70±0.03	0.08
Myocardial infarct uptake	1.05±0.37	1.34±0.22	0.70±0.14	–	<0.001
Remote myocardial uptake	0.80±0.18	0.85±0.17	0.64±0.12	–	0.009
SUV_max_ (kBq/mL)					
Total LV uptake	2.71±0.58	2.86±0.56	2.24±0.40	2.07±0.31	<0.001
Myocardial infarct uptake	3.53±1.00	3.98±0.68	2.18±0.35	–	<0.001
Remote myocardial uptake	2.59±0.64	2.75±0.65	2.18±0.35	–	0.02
TBR_max_					
Total LV uptake	0.98±0.19	1.02±0.19	0.89±0.20	0.81±0.09	0.01
Myocardial infarct uptake	1.28±0.34	1.42±0.25	0.86±0.16	–	<0.001
Remote myocardial uptake	0.94±0.19	0.97±0.20	0.84±0.13	–	0.11
SUV_C_ (kBq/mL)					
Total LV uptake	−0.55±0.57	−0.52±0.55	−0.61±0.67	0.81±0.42	0.85
Myocardial infarct uptake	0.75±0.91	1.13±0.67	−0.40±0.46	–	<0.001
Remote myocardial uptake	−0.19±0.52	−0.11±0.54	−0.45±0.37	–	0.12

*Analysis of variance, Student’s t-test (continuous data) or χ^2^ test (categorical data).

Mean±SD or number (percentage).

CMR, cardiac magnetic resonance; ECV, extracellular volume; LGE, late gadolinium enhancement; LV, left ventricular; LVEDV, LV end diastolic volume; LVEF, LV ejection fraction; LVESV, LV end systolic volume; PET, positron emission tomography; SUV, standardised uptake value; SUV_C_, corrected SUV; SUV_max_, maximum SUV; SVC, superior vena cava; TBR, tissue-to-background ratio; WMI, wall motion index.

Seventeen patients from the acute MI group received follow-up CMR imaging 287±37 days after MI. During this timeframe, there were improvements in LVEF (p<0.01) and regional wall motion (WMI; p<0.005). In total, 43 of 272 myocardial segments (16%) showed an improvement in regional wall motion, while 226 segments remained unchanged and three segments displayed functional deterioration. Infarct size and LV mass also improved (p=0.03 and p<0.01 respectively, table 3).

**Table 3:** Acute MI assessments

	Imaging data		
CMR imaging	Initial CMR (n=21)	Follow-up CMR (n=17)	p Value
MI to CMR (days)	13±5	287±37	<0.01
LVEF (%)	52±9	55±8	<0.01
Indexed LV mass (g/m^2^)	85±20	74±13	<0.01
LVEDV (mL/m^2^)	80±18	82±16	0.88
LVESV (mL/m^2^)	39±14	38±12	0.36
Wall motion index	0.40±0.20	0.22±0.15	<0.01
ECV (%)	34±4	33±2	0.58
Infarct size (g/m^2^)	13 (7–17)	6 (3–14)	0.03
PET imaging			
	Initial PET/CT (n=21)	Repeat PET/CT (n=17)	
MI to PET (days)	12±4	76±19	<0.01
Total LV uptake (TBR_mean_)	0.82±0.18	0.85±0.18	0.96
Myocardial infarct uptake (TBR_mean_)	1.34±0.22	1.20±0.21	0.02
Remote myocardial uptake (TBR_mean_)	0.85±0.17	0.82±0.15	0.38
Segmental uptake (TBR_mean_) and regional WMI			
Normal function (0)	0.80±0.26	0.83±0.23	0.14
Mild-mod hypokinesia (1)	0.89±0.33	0.97±0.29	0.33
Severe hypokinesia (2)	0.97±0.28	0.91±0.34	0.47
Akinesia (3)	0.77±0.21	0.73±0.24	0.66
Dyskinesia (4)	–	–	–
Segmental uptake and transmurality of MI (TBR_mean_)			
No infarct	0.75±0.23	0.81±0.23	NA
Subendocardial infarct (1–75%)	0.95±0.29	1.08±0.28	NA
Transmural infarct (76–100%)	0.89±0.29	0.88±0.27	NA

Mean±SD or median (IQR).

CMR, cardiac magnetic resonance; ECV, mean extracellular volume; LV, left ventricle; LVEDV, LV end diastolic volume; LVEF, LV ejection fraction; LVESV, LV end systolic volume; MI, myocardial infarction; PET, positron emission tomography; TBR_mean_, mean tissue-to-background ratio; WMI, wall motion index.

### Histology and dynamic myocardial ^18^F-fluciclatide PET

**Figure 1:**
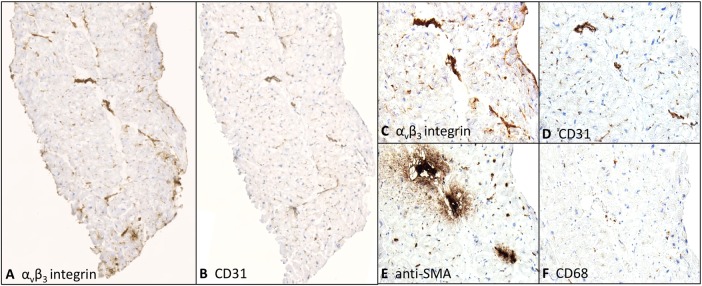
α_v_β_3_ integrin expression in patient with recent myocardial infarction (MI). Adjacent fresh-frozen and cryosectioned biopsies from the peri-infarct area of a patient with recent anterior MI are shown. Immunohistochemical staining for (A) α_v_β_3_ integrin displayed multiple regions of positive staining that co-localise to regions of staining for vascular endothelial cells (CD31), (B) visible at ×4 magnification. At ×20 magnification, these regions of α_v_β_3_ staining (C) correspond predominantly to arterioles and the microvasculature (D) and also to regions of staining for smooth muscle actin (SMA) (E), representative of both arterioles (co-staining with CD31) and myofibroblasts. There were relatively few macrophages (F).

In an exploratory analysis, two patients scheduled for coronary artery bypass surgery within 14 days of infarction underwent myocardial biopsies from the peri-infarct zone (figure 1). This showed predominantly viable myocardium with widespread positive staining for α_v_β_3_ integrin, largely in regions that co-localised to CD31-positive vascular endothelial cells. Interestingly, these sites represented mainly angiogenic microvasculature, although there was scattered co-localisation with dual smooth muscle actin- and CD31-positive arterioles. There were lesser numbers of CD68-positive inflammatory cells and smooth muscle actin-positive myofibroblasts, but where present, these also co-registered with α_v_β_3_ integrin expression (figure 1).

**Figure 2:**
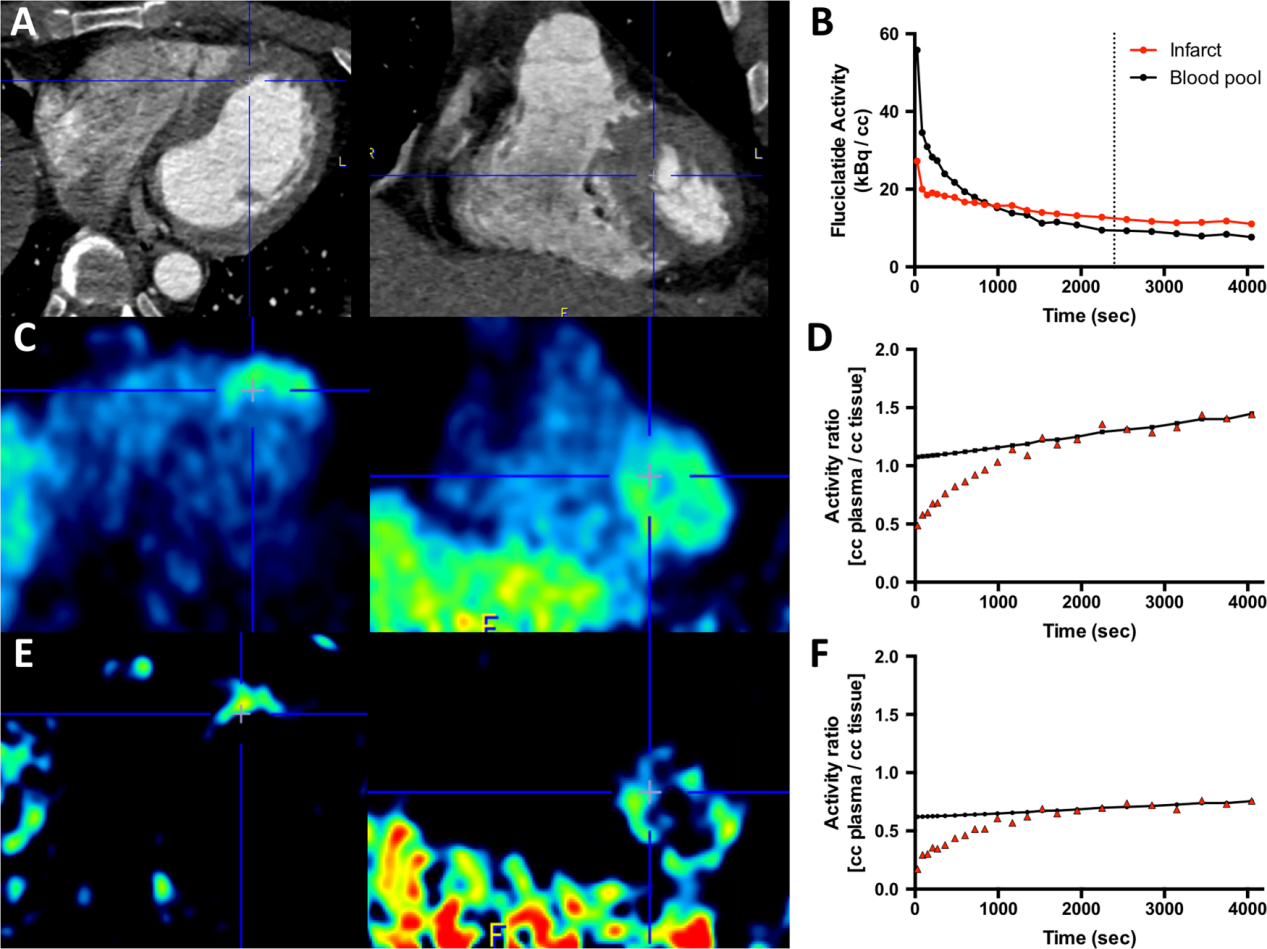
Dynamic analysis of ^18^F-fluciclatide uptake is shown. Axial and sagittal CT angiographies of the thorax in a patient with recent anterior myocardial infarction (A) are shown. The time-activity curves generated from the descending aorta and the apical interventricular septum (blue crosshairs) show increased uptake in the infarct relative to blood pool. Optimal contrast between ^18^F-fluciclatide tissue and blood pool activity was observed after 40 min (dotted line, B). The positron emission tomography image in the axial and sagittal plane shows increased uptake within the apical septum, although there is some background activity (C). Patlak analysis of regions of interests placed in the interventricular septum confirms integrin binding, as evidenced by the gradient of the slope and the y-intercept (D) and, using a *K*i-generated image, we can better identify and delineate focal uptake within myocardium (E). A region of remote myocardium within the same patient generates a Patlak curve with significantly lower gradient and intercept in comparison (F).

Dynamic PET studies were performed in a subgroup of 10 patients. ^18^F-Fluciclatide activity within the region of MI increased gradually and reached a plateau at around 30–40 min (figure 2). The injected activity was cleared from the blood pool with a half-life of about 10 min, so that it remained relatively high during the period of PET acquisition (superior vena cava SUV_mean_ 2.73±0.51 at 40–70 min post-injection). The optimum contrast between ^18^F-fluciclatide uptake in the site of infarction and the blood pool was observed at 40 min (figure 2B). This time point was therefore used for subsequent static imaging.

**Figure 3:**
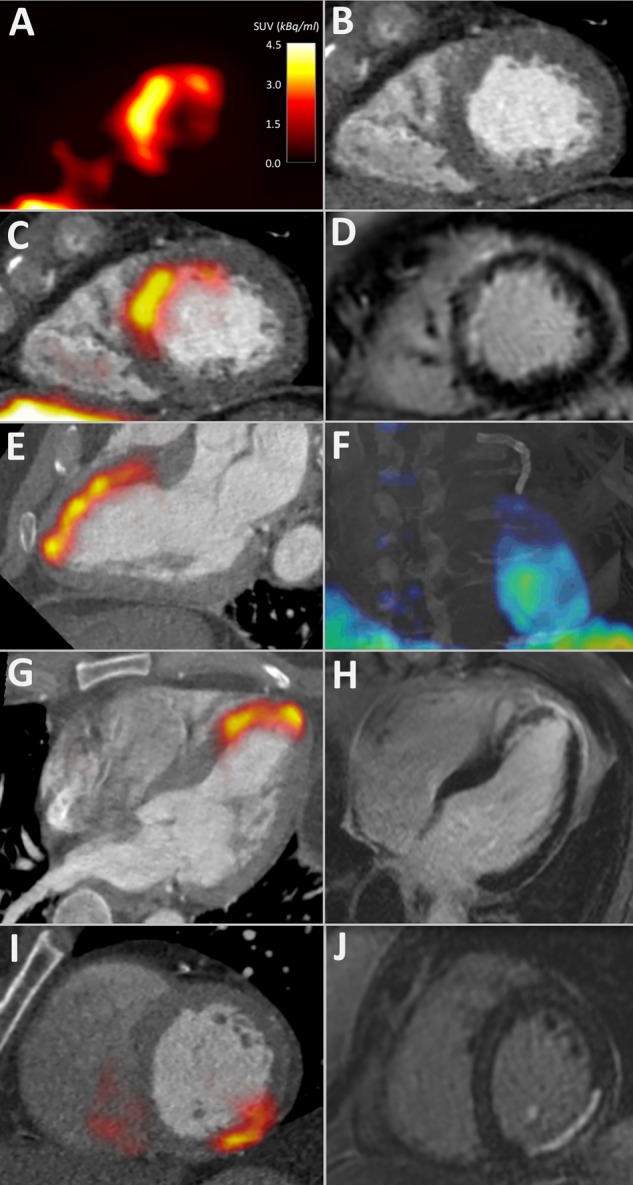
^18^F-Fluciclatide uptake in acute myocardial infarction (MI) is shown. 18F-Fluciclatide uptake in three patients with recent subendocardial MI is shown. Patient 1, 13 days after anterior MI, displaying a short-axis positron emission tomography (PET) image of the left ventricle with crescentic 18F-fluciclatide uptake (A) that correlates with the interventricular septum and anterior wall on CT angiography (B). The fused PET/CT-angiography image (C) shows this uptake to correspond exactly with the region of late gadolinium enhancement (LGE) on cardiac magnetic resonance (CMR) (D). Further delineation of myocardial uptake on PET/CT is clearer in the two-chamber view (E) and on a fused CT/three-dimensional-Patlak image, which shows this uptake to follow a watershed-pattern emerging from the coronary stents present in the left anterior descending coronary artery (F) (see online supplementary video file 1). (G) and (H) Patient 2, 8 days following anterior MI, displaying focal uptake of 18F-fluciclatide in the anterior wall and apex in the three-chamber view on PET/CT (G) which corresponds to the region of infarction on LGE CMR imaging (H). (I) and (J) Patient 3, showing focal uptake of 18F-fluciclatide in the inferior wall 19 days following MI on PET/CT (I) that again corresponds to the infarction on CMR LGE imaging (J).

Using a two-compartment Patlak model,^5^ 18F-flucicatide uptake displayed a distinct linear phase and a steep *K*i slope, in keeping with irreversible binding of ^18^F-fluciclatide to α_v_β_3_ integrin during the 70 min period of evaluation (figure 2D). The three-dimensional parametric images generated from Patlak analysis (figures 2E and 3F and see online supplementary video 1) confirmed regions of increased ^18^F-fluciclatide binding in sites of acute infarction, supporting an upregulation of α_v_β_3_ integrin within the infarct zone.

10.1136/heartjnl-2016-310115.supp2 supplementary video 1

### Static ^18^F-fluciclatide PET in MI

Using static PET images, the interobserver reproducibility assessing ^18^F-fluciclatide uptake in the myocardium was good (see online supplementary table S2). Briefly, quantifying focal uptake in acute infarction was most reproducible using the TBR_mean_ method, showing no fixed or proportional biases (mean % difference 3.0 (95% CI −24.0 to 29.9)) and a high ICC value (0.94 (95% CI 0.83 to 0.98)). This measure was therefore preferentially used for subsequent analysis. Quantification of the regional ^18^F-fluciclatide TBR_mean_ in individual myocardial segments also proved reproducible with no fixed or proportional biases (mean % difference −8.97 (95% CI −31.6 to 13.6)) and a high ICC value (0.90 (95% CI 0.590 to 0.975), see online supplementary table S3).

All patients in the acute MI cohort had increased focal myocardial uptake of ^18^F-fluciclatide on baseline PET/CT scanning (12±4 days after MI), which co-localised to regions of infarction on CMR (figures 2 and 3, tables 2 and 3, see online supplementary videos 1 and 2). Hepatic uptake of ^18^F-fluciclatide was common. However, in the patient with inferior MI, basal inferior and inferoseptal myocardial uptake of ^18^F-fluciclatide was quantifiable. ^18^F-Fluciclatide uptake was greater in the acute myocardial infarct when compared with regions of old infarction in the CTO cohort and healthy myocardium in the control group, regardless of the measure of PET uptake used (eg, TBR_mean_ 1.34±0.22 vs 0.70±0.14 vs 0.70±0.10, p<0.001 respectively). Indeed, no focal increase in ^18^F-fluciclatide uptake was observed in regions of chronic infarction, with similar PET uptake measurements compared with control subjects (eg, TBR_mean_, p=0.83). In regions of myocardium remote to the site of acute infarction, ^18^F-fluciclatide activity was modestly but uniformly increased when compared with comparative regions in patients with CTO (TBR_mean_ 0.85±0.17 vs 0.64±0.12, p=0.009). Across our population, there were no age-related (TBR_mean_, r=−0.19 (−0.53–0.19), p=0.31) or sex-related (TBR_mean_ 1.14±0.07 vs 1.25±0.17, p=0.46) differences in ^18^F-fluciclatide uptake.

10.1136/heartjnl-2016-310115.supp3 supplementary video 2

Seventeen subjects agreed to return for a second ^18^F-fluciclatide PET/CT 76±19 days post-MI, with similar results noted. All these patients were clinically stable between the scans. ^18^F-Fluciclatide uptake remained elevated at the site of infarction (MI vs CTO group, TBR_mean_ 1.20±0.21 vs 0.70±0.15 respectively, p<0.001), although the intensity was reduced compared with earlier imaging (p=0.01; figure 3). Increased ^18^F-fluciclatide uptake also persisted in regions of remote myocardium when compared with uptake in patients with CTO (TBR_mean_ 0.82±0.15 vs 0.64±0.12 respectively, p=0.01) and interestingly remained equivalent in terms of intensity compared with the initial PET scan (TBR_mean_ 0.85±0.17 vs 0.82±0.15 respectively, p=0.38).

### Myocardial ^18^F-fluciclatide uptake and cardiac function

**Figure 4:**
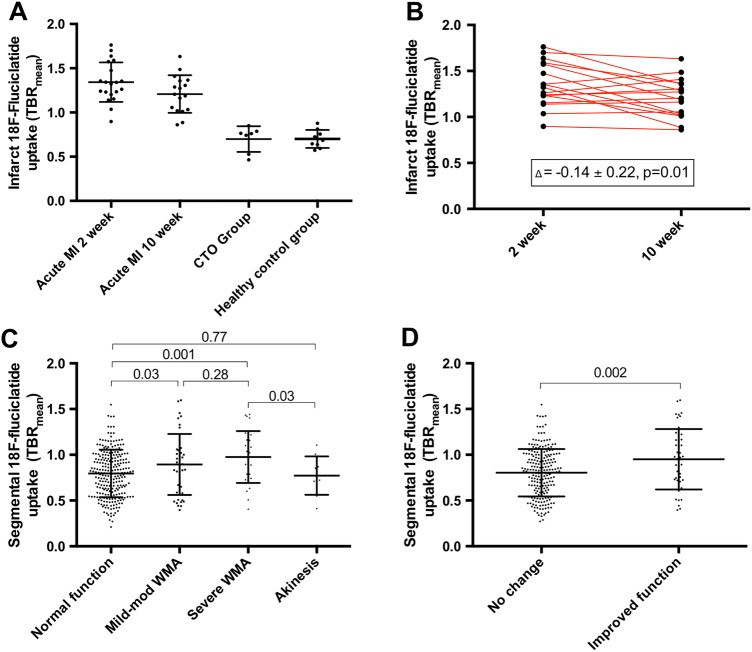
18F-Fluciclatide uptake in myocardial infarction (MI) is shown. Uptake of 18F-fluciclatide in (A) patients with acute MI at 2 and 10 weeks, patients with chronic total occlusion and healthy control subjects is shown. Uptake was greatest at 2 weeks after MI (B). 18F-Fluciclatide uptake in the acute MI group was greater in regions of hypokinesis when compared with sites of normal function or akinesis (C). This translated to a higher 18F-fluciclatide uptake in those regions which subsequently improved in function on follow-up cardiac magnetic resonance (D). CTO, chronic total occlusion; TBR, tissue-to-background ratio. WMA, wall motion abnormality.

The extent of ^18^F-fluciclatide uptake following MI was compared with clinical and imaging measures of MI severity and subsequent repair (see online supplementary table S3). Although segmental ^18^F-fluciclatide uptake displayed a moderate correlation with the degree of ECV on CMR T1 mapping (r=0.37, p<0.001), it did not correlate with many of the standard measures of infarct severity; in particular, there were no associations with infarct size on CMR (r=0.03, p=0.90), LVEF (r=−0.08, p=0.72), hs-cTnI (r=0.13, p=0.36) or C reactive protein (r=−0.20, p=0.38) (see online supplementary table S3). This may be explained by the absence of increased uptake in the largest akinetic infarcts (normal wall motion vs akinetic segments; TBR_mean_ 0.80±0.26 vs 0.77±0.21, p=0.77). Rather, ^18^F-fluciclatide activity was highest in segments with hypokinesis (WMI 1&2 vs 0; TBR_mean_ 0.92±0.03 vs 0.80±0.26, p<0.001; figure 4) and segments associated with a subendocardial pattern of LGE (subendocardial LGE vs no LGE; TBR_mean_ 0.95±0.06 vs 0.75±0.03 respectively, p<0.001; figure 4).

^18^F-Fluciclatide uptake was higher in hypokinetic regions that subsequently demonstrated functional recovery compared with regions with no change or a worsening in contractile function (TBR_mean_ 0.95±0.33 vs 0.81±0.02 respectively, p=0.002; figure 4, see online supplementary video 2). Using logistic regression analysis, ^18^F-fluciclatide TBR_mean_ emerged as a predictor of recovery in segmental cardiac function on univariable analysis (OR 1.27 (95% CI 1.08 to 1.50) per 10% increase in TBR_mean_, p=0.003), which persisted after adjustment for age and sex (OR 1.27 (95% CI 1.07 to 1.51) per 10% increase in TBR_mean_, p=0.005). This effect was however attenuated following additional adjustment for the transmurality of LGE (OR 1.03 (95% CI 0.83 to 1.26) per 10% increase in TBR_mean_, p=0.80) and ECV (OR 1.02 (0.91 to 1.32) per 10% increase in TBR_mean_, p=0.35).

## Discussion

Using the novel radiotracer ^18^F-fluciclatide, we have for the first time described the temporal expression of myocardial α_v_β_3_ integrin receptor in patients with recent acute MI. We demonstrate intense early uptake attributable to regions of recent infarction, in particular subendocardial, hypokinetic infarcts that appeared to demonstrate subsequent functional recovery. Our data suggest that α_v_β_3_ integrin receptor expression can be readily quantified in the infarct zone and that ^18^F-fluciclatide PET may hold promise as a clinical biomarker of healing activity with application to novel pharmacological or cell-based therapies aimed at improving outcome after MI.

Expression of α_v_β_3_ integrin by vascular endothelial cells facilitates myocardial salvage through angiogenesis in the peri-infarct zone, while also mediating the activated macrophage response to inflammatory signals and governing myofibroblast differentiation through the activation of latent transforming growth factor-β1.^9–11^ The α_v_β_3_ integrin contains a binding site for an RGD peptide subunit (the arginine-glycine-aspartate motif) and this is the target for a number of molecular imaging probes. In murine and human studies of acute MI, RGD-based radiotracers accumulate at the site of infarction as early as 3 days, peaking at 1–3 weeks post-MI^4^ ^12^ and correlating with adverse remodelling and infarct scar formation at 12 months.^13^ ^14^ For the first time, we have here confirmed and extended these findings using the highly selective and sensitive PET RGD-radiotracer, ^18^F-fluciclatide. Moreover, given the study sample size and comprehensive imaging assessment, we have been able to make several significant observations as well as incorporate a number of important controls and comparisons.

We have compared ^18^F-fluciclatide uptake in patients with recent acute MI with healthy control subjects and patients with established infarction. We have demonstrated that ^18^F-fluciclatide uptake is specific for acute infarction and does not bind to old established infarcts. Perhaps more importantly, we did not see a correlation with acute infarct size, quantified as the mass of LGE on CMR. Indeed binding of ^18^F-fluciclatide in akinetic infarcts was relatively low and was instead highest in subendocardial infarcts associated with hypokinesia. This would suggest that ^18^F-fluciclatide uptake is not a surrogate of infarction but relates more to the tissue-healing response to injury.

There are a number of potential explanations for the preferential binding of ^18^F-fluciclatide to subendocardial infarcts. It is possible that microvascular obstruction in larger infarcts prevented tissue penetration by ^18^F-fluciclatide or that the presence of tissue necrosis resulted in loss of tissue architecture. However, a number of our observations suggest that ^18^F-fluciclatide uptake reflects novel α_v_β_3_ integrin expression due to re-endothelialisation and angiogenesis in the peri-infarct zone. First, *ex-vivo* histological examination 2 weeks following MI demonstrated that α_v_β_3_ integrin expression predominantly co-localises within endothelial cells of the microvasculature. Second, increased ^18^F-fluciclatide uptake was associated with functional recovery of hypokinetic infarcts. Third, we did not observe ^18^F-fluciclatide uptake in patients with CTO who have chronic and well-established collateral vasculature. Fourth, we did not observe large regions of microvascular obstruction on CMR imaging. This suggests that only newly forming vessels or repopulation of vessels with endothelial cells will result in α_v_β_3_ integrin expression and ^18^F-fluciclatide uptake. This is consistent with similar observations in other diseased states such as angiogenesis associated with malignancy.^5^ ^7^ In our patients with MI, intense binding was observed in the peri-infarct regions of subendocardial MIs with only poorly defined uptake in the central necrotic area. Given these associations and the likelihood that ^18^F-fluciclatide identifies areas of re-endothelialisation and angiogenesis, it is perhaps not surprising that ^18^F-fluciclatide uptake appeared to localise to segments that demonstrated subsequent functional recovery. Taken together, our data suggest that assessing α_v_β_3_ integrin expression in the acute phase of repair might be of use in investigating the healing processes that occur following acute MI. Larger studies are required to confirm these initial findings and assess whether ^18^F-fluciclatide PET provides incremental benefit to CMR (our study was not sufficiently powered). However, as a marker of activity, ^18^F-fluciclatide may be of particular use in assessing the effects of novel therapies aimed at accelerating repair post-MI.

At 10 weeks following MI, ^18^F-fluciclatide uptake persisted in the region of infarction but was reduced compared with the 2-week assessment. This delayed phase of repair is characterised pathologically by a reduction in inflammation and angiogenesis and a more moderated reorganisation of the ECM through myofibroblast-driven type I and type III collagen production.^15^ From our study, we are unable to determine whether this reduction in ^18^F-fluciclatide uptake reflects the waning of re-endothelialisation and angiogenesis or represents a switching to myofibroblast cell types indicative of an enhanced fibrotic response. There was some heterogeneity in the time course of ^18^F-fluciclatide uptake since uptake increased at 10 weeks in six subjects. However, we detected no adverse effects of this increase in uptake and there was no corresponding increase in LGE or ECV on T1 mapping.

Modification of the ECM following MI is limited to the site of infarction. Indeed, the myofibroblast-driven fibrotic expansion seen in the remote myocardium influences global myocardial recovery.^16^ Expression of α_v_β_3_ integrin in remote myocardial regions has been reported up to 6 months following MI.^10^ ^17^ In keeping with this, ^18^F-fluciclatide activity was consistently increased in the remote myocardium at both 2 and 10 weeks when compared with comparative myocardial regions in patients with CTO and healthy controls.

There are some limitations in our study that we should acknowledge. First, despite accounting for systolic motion using ECG gating, cardiac PET is also limited by respiratory motion and this may affect sensitivity in particular due to the high activity in the closely adjacent blood pool and hepatic tissue. This is likely to be a particular problem for inferior infarcts (present in only one of our study subjects), although these less commonly lead to adverse remodelling and heart failure. Novel motion tracking may in the future help to negate some of these issues and enable even greater definition of regional α_v_β_3_ integrin expression.^18^ Second, limited cardiac tissue was available for histological assessment, preventing complete stoichiometric and temporal assessment of α_v_β_3_ integrin expression in humans post-MI. Fortunately, inference can be drawn from extensive animal models of infarction but, for future application of ^18^F-fluciclatide, a more extensive histological assessment would be preferred. Third, our study was not powered to address the incremental value of ^18^F-fluciclatide PET over established predictor markers of cardiac recovery such as CMR. This will require larger patient populations. Instead this study provides the first description of increased ^18^F-fluciclatide in the myocardium following MI, indicating that it provides important information about the LV remodelling response. Further studies will be required to establish the clinical utility of this approach.

In conclusion, we report the largest and most comprehensive analysis of an α_v_β_3_ integrin radiotracer in the assessment of myocardial repair following acute MI. We have demonstrated that increased ^18^F-fluciclatide uptake occurs at sites of acute MI, in particular regions of subendocardial infarction and hypokinesia associated with subsequent functional recovery. Our data suggest that myocardial α_v_β_3_ integrin expression represents a marker of ongoing cardiac repair and that ^18^F-fluciclatide is a potentially useful imaging biomarker for investigating this healing response post-MI.

**Key messages**

**What is already known on this subject?**

- Non-invasive imaging of myocardial remodelling may permit understanding, prediction and potential modification of adverse remodelling and the syndrome of delayed heart failure following myocardial infarction (MI). The α_v_β_3_ integrin cell surface receptor is intrinsic to angiogenesis, inflammation and fibrogenesis in remodelling myocardium and has been targeted using positron emission tomography (PET) radiotracers in murine and small human studies following MI. However, the role of α_v_β_3_ integrin radiotracers in humans following MI is incompletely defined.

**What might this study add?**

- Using PET imaging with kinetic analysis, CT contrast angiography and cardiac MRI, we have demonstrated that ^18^F-fluciclatide binds with α_v_β_3_ integrin receptors in regions of acute MI and in the remote myocardium. ^18^F-fluciclatide uptake correlates with functional impairment and may predict myocardial recovery.

**How might this impact on clinical practice?**

- Our study fulfils a key step validating α_v_β_3_ integrin receptor imaging in humans following MI. This novel characterisation of myocardial remodelling may hold potential as a biological end point in the study of novel therapies following MI.
