# Supplementary material for: Cardiac αVβ3 integrin expression following acute myocardial infarction in humans
Source: Heart. 2016 Dec 7;103(8):607–15. doi: 10.1136/heartjnl-2016-310115 (PMC5566089; doi:10.1136/heartjnl-2016-310115)
Supplement: supplementary data [file heartjnl-2016-310115supp001.pdf]

Supplementary Material

Imaging Assessments

Positron Emission Tomography / Computer Tomography Imaging

All patients underwent PET-CT imaging of the thorax with a hybrid scanner (Biograph mCT, Siemens Medical Systems, Erlangen, Germany) at the Clinical Research Imaging Centre, University of Edinburgh. Subjects were administered a target dose of 230 MBq 18F-fluciclatide. An attenuation-correction CT scan (non-enhanced 120 kV and 50 mA, 3-mm slices) was performed, followed by the PET acquisition with electrocardiographic (ECG) gating. To assess tracer pharmacodynamics and the optimum timing of scanning, dynamic PET imaging of the thorax was initially performed in 10 subjects in 3-dimensional mode with a single bed position for 70 min. The remainder of study subjects underwent static imaging performed at the optimal time point (40 min post-injection) using a single 30-min bed position in list mode. To enable an accurate definition of cardiac anatomy cardiac CT angiography was performed on the hybrid scanner immediately after the PET acquisition: 330 ms rotation time, 100 (body mass index <25 kg/m^2) or 120 (body mass index >25 kg/m^2) kV tube voltage, 160-245 mAs tube current, 3.8 mm/rotation table feed, prospective (heart rate regular and <60 /min), or retrospective (heart rate >60 /min) electrocardiogram-gated. Depending on the body mass index, a bolus of 80-100 mL contrast (400 mgI/mL; Iomeron, Bracco, Milan, Italy) was injected intravenously at 5 mL/s, after determining the appropriate trigger delay with a test bolus of 20 mL contrast material.

PET Kinetic Analysis

Kinetic analysis was undertaken to investigate the uptake of 18F-fluciclatide within the myocardium. The dynamic PET data were reconstructed (Ultra-HD, 2 iterations, 21 subsets, 256 pixels, 1.6-mm pixel size) using a dynamic protocol without ECG gating in following time frames; 60s x 5, 120s x 5, 180s x 5, 300s x 8. Regions of interest (ROI's) were drawn in the descending aorta blood pool and myocardium, and used to derive time activity curves after decay correction. An input function calculation based on the PET image–derived activity curve from the aorta blood pool [1] and the myocardial time-activity curve were used to estimate the tissue influx rate *K*i (the slope of the linear regression) and the volume of distribution (the *y*-axis intercept) using a 2-tissue irreversible Patlak model [2,3]. Thoracic 18F-fluciclatide dynamic activity was then normalized for the blood-pool input function on a voxel-by-voxel basis, and after 3D Gaussian filtering (5-mm FWHM) a parametric 3-dimensional image of 18F-fluciclatide uptake was generated (PMod version 3.409, Pmod technologies limited, Switzerland). Using this image, regions of 18F-fluciclatide binding in the myocardium were identified and manually delineated for subsequent *K*i analysis.

Static Image Reconstruction and Analysis

For all patients, static ECG-gated PET images were reconstructed in diastole (40-70 min post-injection, 50–75% of the R-R interval, Ultra-HD, 2 iterations, 24 subsets, zoom x2, 200 pixels). Images were analysed by an experienced observer (WJ) using an OsiriX workstation (OsiriX version 6.0 64-bit; OsiriX Imaging Software, Geneva,

Switzerland). PET images were fused and aligned with CT angiography datasets in diastole. Myocardial radiotracer uptake was quantified using two methods. First, using a standardized approach PET/CT datasets were re-orientated into traditional short-axis, 2-chamber, 3-chamber and 4-chamber views with a slice thickness of 3 mm in order to fully visualize myocardial radiotracer uptake and allow comparison with CMR imaging. Regions of interest (ROIs) were drawn at sites that corresponded to the areas of acute infarction seen on CMR late gadolinium enhancement imaging. To define referent 'remote' myocardial regions, ROI's were drawn within proximal regions of the myocardial territory that displayed no CMR evidence of infarction. Care was taken to avoid blood-pool contamination. ROIs were copied onto the PET dataset and mean radiotracer activity measured using standard uptake values (SUV; the decay corrected tissue concentration of the tracer divided by the injected dose per body weight) and corrected for radiotracer blood-pool activity in the superior vena cava (SVC) to provide a mean tissue-to-background ratio (TBR_{mean}).[4,5] Second, the CT angiography dataset was re-orientated into the left ventricular short axis (slice thickness 8 mm). Basal, mid-cavity, and apical regions were manually delineated into segmental ROI's according to the standard 17-segment model recommended by the American College of Cardiology/American Heart Association.[6] We excluded the true apex as it was not possible to avoid partial volume effects. ROI's were then copied onto the re-orientated PET image dataset and segmental SUV and TBR data extracted using the technique above. In a substudy of 10 randomly selected subjects, interobserver reproducibility was assessed by two experienced observers (WJ,CM).

MRI Imaging

Cardiac MRI was performed at 3 T (MAGNETOM Verio, Siemens AG, Healthcare Sector, Erlangen, Germany). For the assessment of left ventricular function, short-axis cine images from the mitral valve annulus to the apex were obtained using a balanced steady-state free-precession sequence (8-mm parallel slices with 2-mm spacing). Quantification of left ventricular function and volumes indexed to body surface area was assessed with dedicated software (Siemens AG Healthcare Sector, Erlangen, Germany). Regional systolic function assessments were performed from the basal, mid, and apical short-axis slices by calculating the end-diastolic and end-systolic wall thicknesses and expressed as the wall motion score index (WMSI; 0, normal; 1, mild or moderate hypokinesia; 2, severe hypokinesia; 3, akinesia; 4, dyskinesia).[7] The assessment of focal replacement myocardial fibrosis was performed with late gadolinium enhancement (LGE) imaging, 15 min after administration of 0.1 mmol/kg gadobutrol (Gadovist/Gadavist, Bayer Pharma AG, Berlin, Germany). An inversion recovery fast gradient-echo sequence was applied to the left ventricular short-axis stack with the inversion time optimized to achieve satisfactory nulling of the myocardium. The amount of LGE was quantified with QMASS software (Medis Medical Imaging Systems, Leiden, the Netherlands) using a signal intensity threshold greater than twice the standard deviation above the mean value in a normal region of myocardium sampled on the same short-axis image. The transmural extent of infarction within each segment was classified using a transmurality score (transmurality index; 0, no LGE; 1, 1-50%; 2, 51-75% or 3, 76-100%) and recorded as either subendocardial (1-2) or transmural (3).[8]

Areas thought to represent inversion artefact or blood pool contamination were manually excluded. Myocardial extracellular volume fraction (ECV) has been demonstrated to act as a measure of myocardial fibrosis in a variety of cardiac conditions.[9,10] Recently, our group has described a highly reproducible standardized approach to analyze myocardial ECV. [11] Briefly, myocardial T1 mapping was performed in the mechanism cohort using the modified look-locker inversion recovery sequence: flip angle, 35°; minimum TI, 100 ms; TI increment, 80 ms; and time delay, 150 ms with a heartbeat acquisition scheme of 3-3-5. [12] Regions of interest were drawn around the myocardium on the short-axis, pre-contrast, motion-corrected myocardial T1 maps and copied onto corresponding 20-min post-contrast maps, with minor adjustments made to avoid partial volume effects and artifact (OsiriX version 4.1.1, Geneva, Switzerland). ECV was calculated according to the following formula:

$$ECV=(\Delta R1myocardium/\Delta R1blood\text{-}pool)\times(1-hematocrit)$$

where:

$$\Delta R1=(1/postcontrast\ T1-1/precontrast\ T1).$$

Hematocrit was sampled at the time of MRI. [13]

Histological Assessment

For histological analysis, myocardial biopsy samples were obtained from patients undergoing coronary artery bypass grafting following myocardial infarction. Patients with recent large ST-elevation myocardial infarction (<14 days, hs-cTnI >10,000 ng/L) were considered for inclusion. A core cardiac biopsy was taken intra-operatively under direct

visualisation by an experienced surgeon from the peri-infarct zone. Samples were fresh frozen and mounted in cryosection medium. The tissue samples were then cut in sequential, longitudinal 4-µm sections at -20 °C and thaw-mounted onto microscope slides. They were dried for 15 min and spray-fixed with neutral buffered formalin. After rinsing in distilled water, sections were stained with hematoxylin-eosin (HE) and van-Gieson (VG) for conventional histopathological examination. In order to optimize immunohistochemistry, an antigen-unmasking step was performed by microwave treatment for 30 s. Endogenous peroxidase was blocked by incubation with hydrogen peroxide for 5 min. Sections were subsequently incubated with the primary antibodies; smooth muscle actin, CD31, CD68 (clone PG-M1), and integrin $\alpha_v\beta_3$ antibody, clone LM609 (Millipore) for 30 min at room temperature. After washing the sections were incubated with Envision Flex (DAKO, K5007) for 30 min at room temperature, followed by incubation with diaminobenzamine (Sigma) for 10 min. The slides were finally counterstained with hematoxylin and digitally imaged (Axioscan.Z1, Zeiss, UK) before assessment.

PET Repeatability Studies

The reproducibility of 18F-fluciclatide uptake quantification was assessed in both the blood pool and the myocardium in 10 subjects selected at random. Residual blood pool radiotracer activity was quantified within both the SVC and right atrium. While both methods displayed no fixed or proportional biases with narrow limits of agreement and high ICC values of >0.94, the SVC-approach appeared to hold a slight advantage (ICC 0.97 [95% CI; 0.93-0.99]) and this therefore was applied throughout the study to quantify latent radiotracer blood activity (supplementary table 1). Quantification of

radiotracer uptake within the region of myocardial infarction was assessed using the mean Standard Uptake Value (SUV_{mean}), the mean tissue to background ratio (TBR_{mean}) and a novel method subtracting the target tissue mean SUV from the blood pool mean SUV ($SUV_{target – blood pool}$).[14] The SUV_{mean} and TBR_{mean} both proved highly reproducible, displaying no fixed or proportional biases (mean % difference [95% limits of agreement]; 3.0 [-27.2 - 33.3], and 3.0 [-24.0-29.9] respectively) and a high ICC value (0.93 [0.82-0.97] and 0.940 [0.83-0.98] respectively). Selecting repeatable regions of remote myocardium proved less reliable, with wider limits of agreement (-18.9-66.2) and a moderate intra-class coefficient value of 0.60. The TBR_{mean} method was selected for the study to compensate for potential contamination from residual blood pool activity.

To quantify segmental myocardial uptake, a 16-segment model approach using both the TBR_{mean} the SUV_{mean} quantification methods appeared reliable, again with no fixed or proportional bias and reasonable limits of agreement (mean % difference, -8.97 [-31.6-13.6] and -6.7 [-25.3-11.9], respectively) and an excellent ICC of 0.90 and 0.96 respectively (Table 1). The TBR_{mean} approach was selected for the study again to compensate for potential blood pool contamination,

Supplementary Table 1. Characteristics of patients with acute myocardial infarction

**Clinical Data**	**Acute MI Group** (n=21)
Peak cardiac troponin I (ng/L)	50,000 [26,753-50,000]
Percutaneous coronary intervention	20 (95)
Symptom onset to reperfusion (min)	197 [148-342]
Single vessel disease	10 (48)
**Myocardial Infarction Territory**	
Anterior	16 (76)
Lateral	4 (19)
Inferior	1 (5)
**Adverse Outcome**	
TIMI Flow post-PCI <3	1 (5)
Cardiogenic Shock	3 (14)
IABP	2 (10)
Aborted cardiac arrest	2 (10)

Median [interquartile range] and number (%).

Abbreviations; TIMI, trials in myocardial infarction; PCI, percutaneous coronary intervention;

IABO, intra-aortic balloon pulsation

Supplementary Table 2. 18F-Fluciclatide Reproducibility

	Reproducibility Analysis	
	Mean % difference[a]	Intra-class coefficient[b]
**Blood pool assessment (SUV)**		
SVC	3.1 (-9.5–15.6)	0.971 (0.928-0.989)
Right Atrium	-0.46 (-11.7-10.7)	0.943 (0.868-0.975)
**Infarct assessment**		
$\boldsymbol{TBR_{mean}}$		
Whole Ventricle	-8.97 (-31.6-13.6)	0.898 (0.590-0.975)
Infarct	3.0 (-24.0-29.9)	0.940 (0.833-0.978)
Remote myocardium	23.7 (-18.9-66.2)	0.604 (-0.280-0.847)
$\boldsymbol{SUV_{mean}}$		
Whole ventricle technique	-6.7 (-25.3–11.9)	0.957 (0.830-0.989)
Infarct	3.0 (-27.2-33.3)	0.930 (0.819-0.973)
Remote myocardium	21.2 (-11.1-53.7)	0.787 (0.448-0.918)
Infarct:remote myocardium ratio	-18.2 (-60.6-24.1)	0.687 (0.187-0.879)
$\mathbf{SUV_{target\ tissue\ -\ blood\ pool}}$		
Whole Ventricle	24.1 (-19.2–67.3)	0.410 (-0.372-0.698)
Infarct	77.7 (-464.6–620.1)	0.933 (0.814-0.976)
Remote Myocardium	-43.8 (-120.4-32.7)	0.574 (-0.106-0.836)

[a] Mean % difference between standard uptake value (SUV; kBq/mL) measurements (95% limits of agreement), and [b] ICC (intraclass correlation coefficient) values (95% confidence intervals) for 18F-fluciclatide myocardial uptake and residual blood pool activity.

SVC, superior vena cava; TBR_{mean}, mean tissue to background ratio.

Supplementary Table 3; Comparison of 18F-Fluciclatide Uptake and Indices of Infarction

	18F-Fluciclatide Infarct uptake (TBR_{mean})	Δ 18F-Fluciclatide Infarct uptake (1st-2nd scan, TBR_{mean})	Mean ECV (%)	Infarct Size (g/m^2)
**Clinical Characteristics**				
Peak hs-cTnI (ng/l)	*r*=0.13 (-0.31-0.54) p=0.56	*r*=-0.27 (-0.66-0.24) p=0.30	*r*=0.61 (0.23-0.82) p=0.003	*r*=0.59 (0.22-0.81) p=0.004
hs-CRP (mg/L)	*r*=-0.20 (-0.58-0.25) p=0.38	*r*=-0.04 (-0.54-0.48) p=0.90	*r*=-0.02 (-0.45-0.41) p=0.92	*r*=0.55 (0.16-0.80) p=0.009
**Baseline CMR Assessment**				
LVEF (%)	*r*=-0.08 (-0.49-0.36) p=0.72	*r*=0.03 (-0.46-0.50) p=0.91	*r*=-0.12 (0.53-0.32) p=0.59	*r*=-0.44 (-0.73--0.01) p=0.05
LV mass (g/m^2)	*r*=-0.19 (0.57-0.26) p=0.03	*r*=0.28 (-0.23-0.67) p=0.26	*r*=0.18 (-0.27-0.57) p=0.43	*r*=0.52 (0.12-0.78) p=0.01
Infarct size (g/m^2)	*r*=0.03 (-0.41-0.45) p=0.90	*r*=0.07 (-0.43-0.52) p=0.82	*r*=0.47 (0.06-0.75) p=0.03	-
ECV / segment (%)[Ψ]	*r*=0.37 (0.28-0.45) p<0.001	r=0.22 (0.09–0.34) p=0.003	-	-
**Follow-up CMR Assessment (9 months)**				
Δ LVEF (%)	*r* =-0.23 (-0.65-0.30) p=0.39	*r*=-0.37 (-0.75-0.19) p=0.13	*r*=-0.24 (-0.66-0.28) p=0.36	*r*=0.12 (-0.40-0.58) p=0.66
Δ LV mass (g/m^2)	*r*=0.14 (-0.38-0.60) p=0.60	*r*=0.16 (-0.39-0.64) p=0.57	*r*=-0.60 (-0.85--0.16) p=0.01	*r*=-0.35 (-0.72-0.17) p=0.17
Δ LVEDV (mL/m^2)	*r*=0.34 (-0.19-0.71) p=0.20	*r*=-0.47 (-0.80-0.08) p=0.09	*r*=-0.22 (-0.64-0.31) p=0.41	*r*=-0.06 (-0.54-0.45) p=0.83
Δ Infarct size (g/m^2)	*r*=0.25 (-0.28-0.66) p=0.35	*r*=-0.14 (-0.62-0.41) p=0.62	*r*=-0.61 (-0.85-0.16) p=0.01	*r*=-0.42 (-0.76-0.08) p=0.10
Δ ECV / segment (%)	*r*=-0.11 (-0.22-0.01) p=0.07	*r*=-0.33 (-0.70-0.16) p=0.18	-	-

[Ψ] ECV and PET association assessed per segment. Abbreviations: hs-cTnI, high sensitivity cardiac troponin I; hs-CRP, high sensitivity c-reactive protein; LVEF, left ventricular ejection fraction; LV, left ventricular; LVEDV, left ventricular end diastolic volume; ECV, mean extracellular volume

References

1 Mena E, Owenius R, Turkbey B, *et al.* [18F]Fluciclatide in the in vivo evaluation of human melanoma and renal tumors expressing αvβ3 and αvβ5 integrins. *Eur J Nucl Med Mol Imaging* 2014;**41**:1879–88. doi:10.1007/s00259-014-2791-x

2 Kenny LM, Coombes RC, Oulie I, *et al.* Phase I trial of the positron-emitting Arg-Gly-Asp (RGD) peptide radioligand 18F-AH111585 in breast cancer patients. *Journal of Nuclear Medicine* 2008;**49**:879–86. doi:10.2967/jnumed.107.049452

3 Tomasi G, Kenny L, Mauri F, *et al.* Quantification of receptor-ligand binding with [18F]fluciclatide in metastatic breast cancer patients. *Eur J Nucl Med Mol Imaging* 2011;**38**:2186–97. doi:10.1007/s00259-011-1907-9

4 Dweck MR, Jenkins WSA, Vesey AT, *et al.* 18F-NaF Uptake Is a Marker of Active Calcification and Disease Progression in Patients with Aortic Stenosis. *Circulation: Cardiovascular Imaging* Published Online First: 7 February 2014. doi:10.1161/CIRCIMAGING.113.001508

5 Dweck MR, Jones C, Joshi NV, *et al.* Assessment of Valvular Calcification and Inflammation by Positron Emission Tomography in Patients With Aortic Stenosis. *Circulation* 2012;**125**:76–86. doi:10.1161/CIRCULATIONAHA.111.051052

6 Cerqueira MD, Weissman NJ, Dilsizian V, *et al.* Standardized myocardial segmentation and nomenclature for tomographic imaging of the heart. A statement for healthcare professionals from the Cardiac Imaging Committee of the Council on Clinical Cardiology of the American Heart Association. 2002. 539–42.

7 Chan W, Duffy SJ, White DA, *et al.* Acute left ventricular remodeling following myocardial infarction: coupling of regional healing with remote extracellular matrix expansion. *JACC Cardiovasc Imaging* 2012;**5**:884–93. doi:10.1016/j.jcmg.2012.03.015

8 Kim RJ, Wu E, Rafael A, *et al.* The use of contrast-enhanced magnetic resonance imaging to identify reversible myocardial dysfunction. *N Engl J Med* 2000;**343**:1445–53. doi:10.1056/NEJM200011163432003

9 Treibel TA, White SK, Moon JC. Myocardial Tissue Characterization: Histological and Pathophysiological Correlation. *Curr Cardiovasc Imaging Rep* 2014;**7**:9254. doi:10.1007/s12410-013-9254-9

10 Chin CWL, Shah ASV, McAllister DA, *et al.* High-sensitivity troponin I concentrations are a marker of an advanced hypertrophic response and adverse outcomes in patients with aortic stenosis. *European Heart Journal* 2014;**35**:2312–21. doi:10.1093/eurheartj/ehu189

11 Chin CWL, Semple S, Malley T, *et al.* Optimization and comparison of myocardial

T1 techniques at 3T in patients with aortic stenosis. *Eur Heart J Cardiovasc Imaging* Published Online First: 25 November 2013. doi:10.1093/ehjci/jet245

12 Messroghli DR, Greiser A, Fröhlich M, *et al.* Optimization and validation of a fully-integrated pulse sequence for modified look-locker inversion-recovery (MOLLI) T1 mapping of the heart. *J Magn Reson Imaging* 2007;**26**:1081–6. doi:10.1002/jmri.21119

13 Shah ASV, Chin CWL, Vassiliou V, *et al.* Left ventricular hypertrophy with strain and aortic stenosis. *Circulation* 2014;**130**:1607–16. doi:10.1161/CIRCULATIONAHA.114.011085

14 Chen W, DILSIZIAN V. PET Assessment of Vascular Inflammation and Atherosclerotic Plaques: SUV or TBR? *Journal of Nuclear Medicine* 2015;**56**:503–4. doi:10.2967/jnumed.115.154385
